# Supplementary material for: Identification of photoperiod-induced specific miRNAs in the adrenal glands of Sunite sheep (Ovis aries)
Source: Front Vet Sci. 2022 Jul 22;9:888207. doi: 10.3389/fvets.2022.888207 (PMC9354845; doi:10.3389/fvets.2022.888207)
Supplement: Supplementary file 1 [file Data_Sheet_1.ZIP › Supplementary file/miRDeep2 file.zip!/miRDeep2 file/Know_Structure/SPLP42A3/oar-mir-200b.pdf]

miRBase precursor	: oar-mir-200b
Total read count	: 51
oar-miR-200b read count	: 49
remaining reads	: 2

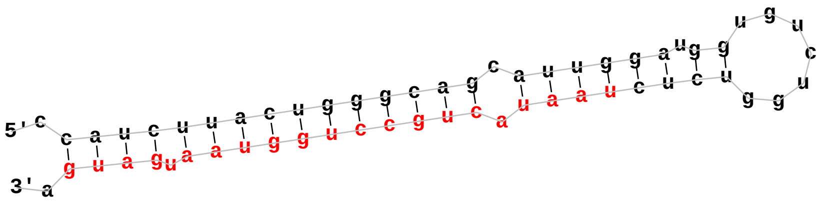

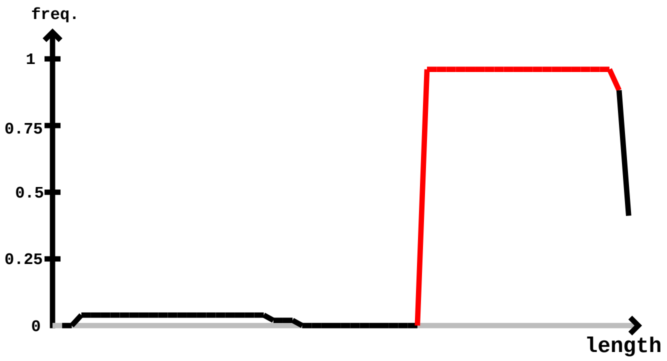


```
                                   oar-miR-200b
5'- ccaucuuacugggcagcauuggauggugucuggucucuaauacugccugguaaugauga   -3'    exp
    .((((((((((((((.((((((.((.......)))))))).))))))))))).)))).    reads      mm         sample
    .caucuuacugggcagcauug.......................................    1          0          seq
    .caucuuacugggcagcauuggau....................................    1          0          seq
    .....................................uaauacugccugguaaugau..    4          0          seq
    .....................................uaauacugccugguaaugaug.    24         0          seq
    .....................................uaauacugccugguaaugauga    21         0          seq
```
